# Effectiveness and safety of adalimumab biosimilar in bio-naive patients with inflammatory bowel disease: a real-life multicenter observational study comparing ABP501, SB5, MSB11022, GP2017, and FKB327

**DOI:** 10.1093/crocol/otag002

**Published:** 2026-01-12

**Authors:** Cristina Regueiro, Maria Teresa Vázquez Rey, Iria Bastón-Rey, Monica Ayude Galego, Amalia Carmona Campos, Alina Montserrat Baz López, Gema Molina Arriero, Maria Jesús Ruiz Barcia, Pablo Vega Villaamil, on behalf of EIGA

**Affiliations:** Research Group in Gastrointestinal Oncology, Galicia Sur Health Research Institute, Ourense, Spain; Gastroenterology Department, Complexo Hospitalario Universitario A Coruña, A Coruña, Spain; Gastroenterology Department, Hospital Universitario Clínico de Santiago, Santiago de Compostela, Spain; Gastroenterology Department, Complexo Hospitalario Universitario de Vigo, Vigo, Spain; Gastroenterology Department, Hospital Ribera Povisa, Vigo, Spain; Gastroenterology Department, Complexo Hospitalario Universitario Lucus Augusti, Lugo, Spain; Gastroenterology Department, Complexo Hospitalario Universitario de Ferrol, Ferrol, Spain; Gastroenterology Department, Hospital da Costa, Burela, Spain; Gastroenterology Department, Complexo Hospitalario Universitario de Ourense, Ourense, Spain

**Keywords:** inflammatory bowel disease, adalimumab, biosimilar, effectiveness, safety

## Abstract

**Background:**

Biosimilars represent a significant opportunity in the treatment of inflammatory bowel disease (IBD). Our aim is to assess the effectiveness and safety of the five approved adalimumab (ADA) biosimilars in IBD patients naive to biologics.

**Methods:**

IBD patients naive to biologics from eight Spanish hospitals were enrolled. We included patients who started ADA biosimilars between November 2018 and January 2022. The study endpoints included (1) induction of remission at week 8; (2) drug persistence at the conclusion of the follow-up period; and (3) safety of the five ADA biosimilars.

**Results:**

In total, 383 patients were included. After induction, 63.8% of patients were in clinical remission. In total, 114 (29.8%) patients discontinued treatment during follow-up. Clinical remission was maintained in 78.4% of patients after a median follow-up of 18 (12-24) months. Dose intensification was performed in 35 (9.1%) patients during follow-up. There was no significant difference in effectiveness for the 5-ADA biosimilars. Additionally, drug persistence was significantly higher in Crohn’s disease (CD) patients (*P* = .012), in the group of patients co-treated with immunomodulators (IMM) (*P* = .001) and in patients with post-induction (at week 8) ADA levels ≥ 7 μg/mL (*P* = .002). Adverse events were reported in 30 (7.8%) patients with no significant difference between ADA biosimilars.

**Conclusion:**

ADA biosimilars are safe and effective in inducing and maintaining remission in a real-life population of bio-naive IBD patients. Furthermore, there is no significant difference between the 5-ADA biosimilars. Drug persistence was significantly higher in patients with CD treated with IMM and with post-induction ADA levels ≥7 μg/mL.

## Graphical abstract

**Graphical Abstract:**
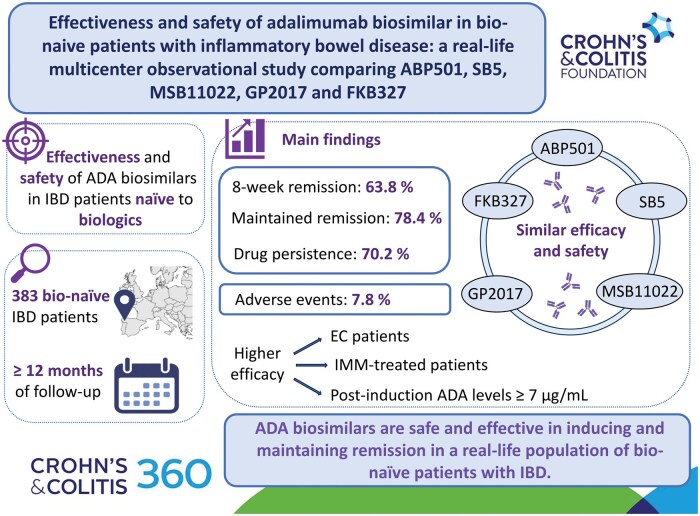

## Introduction

Inflammatory bowel disease (IBD), which includes Crohn’s disease (CD) and ulcerative colitis (UC), is a chronic inflammatory gastrointestinal disease with a recurrent and unpredictable course.^1^^,^^2^ It is a complex disease that manifests mainly at an early age, with increasing incidence and prevalence, and that involves a high consumption of health and economic resources.^1^^,^^3^

Monoclonal antibodies against tumor necrosis factor α (TNFα), such as infliximab (IFX) and adalimumab (ADA), were the first biologic drugs approved for IBD.^4^ Anti-TNFα therapy induces and maintains remission and reduces the rate of surgery and IBD-related hospitalizations.^5^ However, the use of anti-TNFα therapy is costly and has increased the burden of IBD on healthcare systems.^6–8^ In this way, anti-TNFα therapy has been the main cost driver in IBD, accounting for up to 73% of the annual IBD-related healthcare costs.^6^^,^^7^

The patents for the first anti-TNFs approved for use in IBD, IFX, and ADA, have expired, resulting in the emergence of biosimilar drugs. Biosimilars represent a great opportunity in cost saving and allow more patients to access to anti-TNFα therapy.^9^

The first biosimilar to IFX [CT-P13] was approved in 2013, after the publication of two randomized, double-blind studies ­demonstrating its bioequivalence to originator IFX in patients with rheumatoid arthritis and ankylosing spondylitis.^10^^,^^11^ Subsequently, ­numerous prospective and retrospective series have been published supporting its use in patients with IBD.^12^ The first ADA biosimilar arrived later, in 2017, following the publication of two phase III studies in patients with psoriasis and rheumatoid arthritis.^13^^,^^14^ The information on the use and validity of ADA biosimilars in IBD is more limited.^12^ Data on its efficacy and safety in IBD is based on prospective and retrospective studies, mainly evaluating the switch from the original drug.^15–18^ Overall, the results are good and show that ADA biosimilars are not inferior to the originator molecule in terms of efficacy and safety. Still, there are limited data about the safety and effectiveness of ADA biosimilars in patients not previously treated with biologics.^19–21^ Apart from the limited number of studies conducted in bio-naive patients, there is no data available for comparison between the five currently approved ADA biosimilars: ABP 501 (Amgevita, Amgen Inc.), SB5 (Imraldi, Samsung Bioepis UK Limited), MSB11022 (Idacio, Fresenius Kabi Deuschland GmbH), GP2017 (Hyrimoz, Sandoz GmbH), and FKB327 (Hulio, Mylan SAS).^12^ The majority of studies have focused on the safety and efficacy of ABP501 and SB5, particularly in the context of switching from the originator.^15^^,^^17^^,^^19^ A previous study in a cohort of Italian patients has evaluated the efficacy and safety of four of the currently available ADA biosimilars.^21^ However, at this time, data on the effectiveness and safety of all available ADA biosimilars is not yet available. Furthermore, data on the five ADA biosimilars, particularly in IBD patients who have not previously received biologic therapy, is of great importance given the limited number of studies that have been conducted to date. Consequently, the aim of this study was to assess the effectiveness and safety of available ADA biosimilars in bio-naive patients from 8 Spanish hospitals. Additionally, a comparison of the 5 ADA biosimilars in terms of effectiveness and occurrence of adverse effects was conducted.

## Methods

### Study design

We conducted a retrospective, multicenter, observational study. All patients with IBD naive to biologics who started ADA biosimilar between November 2018 and January 2022 in 8 Spanish centers were enrolled. Eligible patients were men and women over 18 years of age with a confirmed diagnosis of UC or CD and who had completed at least induction treatment. Data were collected at baseline, after induction (week 8), and every 6 months. Patients were consecutively included from each participating center without matching or stratification. Exclusion criteria were as follows: patients under 18 years of age; patients who had previously received another advanced therapy such, a different anti-TNF agent, anti-integrin, anti-IL 12/23 or i-JAK, either for the treatment of their IBD or for any other concomitant condition; patients who did not complete induction treatment or those patients who refused to sign the informed consent to participate in the present study. Patients were treated with ADA biosimilars ABP501, SB5, MSB11022, GP2017, or FKB327. ADA biosimilars were given subcutaneously at a dose of 160 mg at week 0, 80 mg at week 2, and then 40 mg every 2 weeks. GP2017 was the most frequently prescribed ADA biosimilar in our cohort, primarily due to logistical factors and pharmacy supply at participating hospitals. The choice of biosimilar was influenced by local hospital procurement processes rather than differences in clinical efficacy or safety*.* We collected the following baseline characteristics: sex, age, medical history, smoking status, body mass index (BMI), subtypes of disease (Montreal classification), disease extent, disease duration, co-treatment with IMM, previous IBD-related surgery, and extraintestinal manifestations (EIMs). The study was conducted following clinical practice guidelines. All patients gave written informed consent.

### Clinical assessment

Disease activity was determined using the Harvey-Bradshaw Index (HBI)^22^ in CD patients and the partial Mayo score (pMS) in UC patients.^23^ Drug persistence and reasons for discontinuation were assessed (primary non-response, secondary loss of response, and adverse event). Dose adjustments, C-reactive protein (CRP), fecal calprotectin (FCAL), ADA drug levels, and anti-drug antibody levels were also collected. ADA levels were measured at post-induction time points, depending on local practice. In our study, ADA trough levels were collected according to the routine clinical practice of each participating center, which could be either proactive or reactive. ADA trough levels and anti-drug antibodies were measured ­locally in the biochemistry departments of each participating hospital using validated assays with comparable cut-offs. This approach ensured that ADA levels were consistent across all centers and minimized potential variability in result interpretation. All clinical parameters were assessed at baseline and during follow-up.

### Outcomes

The primary outcome was to assess the effectiveness and safety of ADA biosimilars in bio-naive IBD patients. Secondary outcomes included a comparison of the 5-ADA biosimilars in terms of effectiveness and safety. The cut-off points for the different determinations were defined using widely accepted thresholds: pMS ≤2 for UC ­patients, HBI ≤4 for CD patients, CRP <0.5 mg/dL and FCAL <250 μg/g. These thresholds are consistent with previous studies and clinical practice and have been shown to correlate with symptomatic remission and disease control.^22^^,^^23^ All outcomes were evaluated at week 8 and at the end of follow-up in terms of the following:

- Drug persistence: persistence on ADA biosimilar at the end of follow-up
- Clinical remission: clinical remission was defined as a pMS ≤2 in UC patients and as a HBI score ≤4 in patients diagnosed with CD.
- Clinical response: clinical response was defined as a decrease in pMS score ≥3 points in UC patients or as a 50% decrease in HBI in CD patients.
- Biochemical remission: biochemical remission was defined as CRP <0.5 mg/dL and FCAL <250 μg/g.
- Primary non-response (PNR): primary failure was defined as no clinical response after the induction period.
- Secondary non-response (SNR): secondary failure was defined as patients who respond to induction but have a loss of response during maintenance leading to discontinuation.
- Dose intensification: intensification was defined as a change from the standard dose of ADA 40 mg every 2 weeks to a dose of 40 mg weekly or 80 mg every 2 weeks.
- Safety: safety was defined as the absence of AEs during treatment.

### AE assessment

AEs were collected throughout the follow-up period. We recorded both, those that led to discontinuation of treatment and those that led to hospitalization of the patient. The occurrence of opportunistic infections was also included as an adverse AE.

### Statistics

Continuous variables were reported as medians and interquartile ranges or means and standard deviations, depending on distribution. Categorical variables were defined as frequencies and ­percentages. The chi-squared test was used to analyze differences between categorical variables. A *t*-test or Mann–Whitney *U*-test was used for comparison between continuous variables. Drug persistence was established with Kaplan–Meier curves. Time-to-event was calculated from the start of ADA biosimilar until drug discontinuation. Patients were censored at the end of follow-up, which was defined as the last gastroenterology-related medical visit. Comparison of treatment persistence between IMM vs non-IMM patients, CD vs UC patients, and ADA levels ≥7 µg/mL vs ADA levels <7 µg/mL was performed by the log-rank test and hazard ratios derived from Cox proportional hazards analysis. *P*-values <.05 were considered to be statistically significant. All statistical analyses were performed using SPSS version 25 (SPSS Inc.).

### Ethical considerations

The present study follows the principles of Declaration of Helsinki, in the Council of Europe Convention on Human Rights and Biomedicine, and according to the Spanish legislation in the field of biomedical research, the protection of personal data, and bioethics. All patients gave written informed consent. Ethics committee approval was obtained from the Clinical Research Ethics Committee of Galicia, Spain (2022/199).

## Results

### Demographic characteristics

In total, 383 patients were enrolled, including 95 with UC and 283 with CD. The baseline characteristics of the study patients are reported in Table 1. Overall, the median duration of follow-up was 18.0 (12.0-24.0) months. There were differences in visit frequency and assessment schedules between biosimilar groups. The median follow-up time for each biosimilar was as follows: GP2017, 12.0 (6.0-18.0) months; ABP501, 30.0 (24.0-42.0) months; SB5, 24.0 (15.0-30.0) months; MSB11022, 12.0 (6.0-18.0) months; and FKB327, 18.0 (12.0-33.0) months. The ADA biosimilar was most commonly prescribed for patients who were refractory to IMM (32.9%), corticodependent (24.8%), following a top-down strategy (12.5%), or for prevention of postsurgical recurrence (6.3%). In UC patients, the primary indication was corticosteroid-dependent (47.4%), while in CD patients, refractory to IMM was the most common indication (37.5%). GP2017 was the biosimilar most frequently used (60.2%), followed by ABP501 (21.5%), MSB11022 (9.4%), SB5 (5.0%), or FKB327 (3.9%). The study revealed no significant differences between UC and CD patients, except for the percentage of women and smoking, which was significantly higher in the CD group (Table 1). Additionally, the percentage of previous surgeries related to IBD was found to be significantly higher in the CD group (30.4% vs 7.4%, *P* <.001) (Table 1).

**Table 1:** Baseline characteristics of the patients.

	Total (*N* = 383)^a^	UC (*N* = 95)	CD (*N* = 283)	*P* value
**Male gender, *n* (%)**	178 (47.8)	55 (58.5)	121 (44.3)	.010
**Age (years), median (IQR)**	47.0 (34.0-59.3)	47.0 (34.0-60.0)	47.0 (34.0-59.3)	.341
**BMI (kg/m²), median (IQR)**	25.2 (22.3-28.7)	25.2 (22.2-28.7)	25.2 (22.3-28.7)	.233
**Disease duration (years), median (IQR)**	3.4 (0.8-10.0)	4.5 (1.4-9.4)	3.0 (0.5-10.7)	.495
**Smoking behaviour, *n* (%)**				
** Never**	166 (46.1)	47 (51.6)	117 (44.3)	.226
** Former**	116 (32.2)	9 (9.9)	69 (26.1)	.001
** Current**	78 (21.6)	35 (38.5)	78 (29.5)	.012
**UC extension, *n* (%)**				
** Proctitis (Montreal E1)**	-	17 (17.9)	-	-
** Left-sided colitis (Montreal E2)**	-	35 (36.8)	-	-
** Extensive colitis (Montreal E3)**	-	43 (45.3)	-	-
**CD location, *n* (%)**				
** Ileal (Montreal L1)**	-	-	160 (56.5)	-
** Colonic (Montreal L2)**	-	-	22 (7.8)	-
** Ileocolonic (Montreal L3)**	-	-	97 (34,3)	-
** Upper gastrointestinal tract (Montreal L4)**	-	-	14 (4.9)	-
**CD phenotype, *n* (%)**				
** Inflammatory (Montreal B1)**	-	-	169 (59.7)	-
** Stricturing (Montreal B2)**	-	-	98 (34.6)	-
** Fistulising (Montreal B3)**	-	-	54 (19.1)	-
**Perianal disease, *n* (%)**	34 (8.9)	0	34 (12.0)	-
**Extraintestinal manifestations, *n* (%)**	70 (18.3)	21 (22.1)	48 (17.0)	.261
**Previous IBD-related surgery, *n* (%)**	94 (24.5)	7 (7.4)	86 (30.4)	<.001
**CRP (mg/dL), median (IQR)**	1.1 (0.4-8.7)	1.1 (0.4-8.9)	1.1 (0.4-8.7)	
**FCAL (mg/dL), median (IQR)**	464.5 (143.5-1139.3)	464.0 (143.0-1107.0)	464.5 (143.5-1139.3)	
**pMS, median (IQR)**	-	5 (3-6)	-	-
**HBI, median (IQR)**	-	-	4 (2-6)	-
**Concurrent immunosuppressant, *n* (%)**	131 (34.2)	26 (27.4)	104 (36.7)	.096

Abbreviations: BMI, body mass index; CD, Crohn’s disease; CRP, C-reactive protein; FCAL, fecal calprotectin; HBI, Harvey-Bradshaw index; IQR, interquartile range; pMS, partial Mayo score; UC, ulcerative colitis.

a Five patients diagnosed with undifferentiated colitis.

### Clinical effectiveness

**Figure 1:**
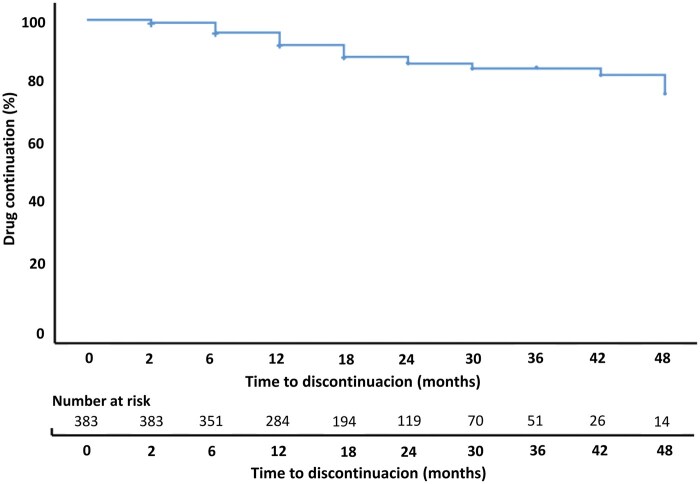
Kaplan-Meier curve of time to ADA biosimilar treatment discontinuation.

After induction, 63.8% of patients were in clinical remission. A proportion of 85.3% of patients achieved a clinical response, and a total of 56.9% of patients achieved biochemical remission. At this time of follow-up (8 weeks), 14 patients discontinued treatment mainly due to PNR (57.1%) and AEs (42.9%). In total, 114 (29.8%) patients discontinued treatment during a median of follow-up time of 18.0 (12.0-24.0) months (Figure 1). The main reasons for stopping therapy were SNR (43.0%), PNR (30.7%), and AEs (26.3%). At the end of the follow-up clinical remission was maintained in 185 (78.4%) patients, and 73.6% of the patients achieved clinical response. Additionally, biochemical remission was maintained in 72.5% of patients.

After induction (8 weeks), clinical remission rates were higher for the biosimilars GP2017 (61.7%), ABP501 (71.6%), and SB5 (73.7%), compared to remission rates for MSB11022 (59.5%) and FKB327 (45.5%). However, statistical comparison showed no significant difference between the remission rates of the 5 biosimilars after 8 weeks of follow-up (*P* = .3). Overall, clinical remission was maintained similarly among the five biosimilars used. It was maintained in 76.6% of patients in the GP2017 group, 79.3% of patients in the ABP501 group, 85.7% of patients in the SB5 group, 86.4% of patients in the MSB11022 group, and 80.0% of patients in the FKB327 group (*P* = .7). At the end of follow-up, no significant differences were found between the remission rates of the 5 biosimilars, nor in the SNR or PNR rates (Table 3). In terms of persistence, all 5 biosimilars showed similar survival at the end of follow-up (Table 3).

**Table 2:** Clinical effectiveness in CD and UC patients at the end of follow-up.

	CD vs UC	*P* value^a^	IMM vs non-IMM	*P* value^b^
**Clinical remission, *n* (%)**	197 (69.6) vs 60 (63.2)	.243	95 (72.5) vs 165 (65.5)	.161
**Clinical response, *n* (%)**	228 (80.6) vs 67 (70.5)	0.041	105 (80.2) vs 195 (77.4)	.532
**Biochemical remission, *n* (%)**	165 (58.3) vs 56 (58.9)	.912	82 (62.6) vs 146 (57.9)	.378
**PNR, *n* (%)**	22 (7.8) vs 13 (13.7)	.086	9 (6.9) vs 26 (10.3)	0.267
**SNR, *n* (%)**	36 (12.7) vs 13 (13.7)	.809	13 (9.9) vs 36 (14.3)	.225

Abbreviations: CD, Crohn’s disease; IMM, immunomodulator treated patients; non-IMM, non immunomodulator treated patients; PNR, primary non-response; SNR, secondary non-response; UC, ulcerative colitis.

a The *P*-value obtained by comparing CD (*n* = 283) vs UC (*n* = 95) patients.

b The *P*-value obtained by comparing IMM (*n* = 131) vs non-IMM (*n* = 252) treated patients.

**Table 3:** Clinical effectiveness among the five ADA-biosimilars used at the end of follow-up.

	Total	GP2017	ABP501	SB5	MSB11022	FKB327	*P* value^a^
	(*N* = 383)	(*N* = 230)	(*N* = 82)	(*N* = 19)	(*N* = 37)	(*N* = 15)	
**Drug persistence, *n* (%)**	269 (70.2)	164 (71.3)	57 (69.5)	15 (78.9)	23 (62.2)	10 (66.7)	.721
**Clinical remission, *n* (%)**	260 (67.9)	159 (69.1)	57 (69.4)	15 (78.9)	21 (56.8)	8 (53.4)	.378
**Clinical response, *n* (%)**	282 (73.6)	186 (80.9)	60 (73.2)	16 (84.2)	25 (67.6)	12 (80.0)	.284
**Biochemical remission, *n* (%)**	216 (56.4)	122 (53.0)	37 (45.1)	11 (57.9)	22 (59.5)	7 (46.7)	.572
**PNR, *n* (%)**	35 (9.1)	21 (9.1)	9 (11.0)	-	3 (8.1)	2 (13.4)	.903
**SNR, *n* (%)**	49 (12.8)	29 (12.6)	9 (11.0)	3 (15.8)	6 (16.2)	2 (13.4)	.351

Abbreviations: PNR, primary non-response; SNR, secondary non-response.

a The *P*-value resulted from comparing the five biosimilars.

**Figure 2:**
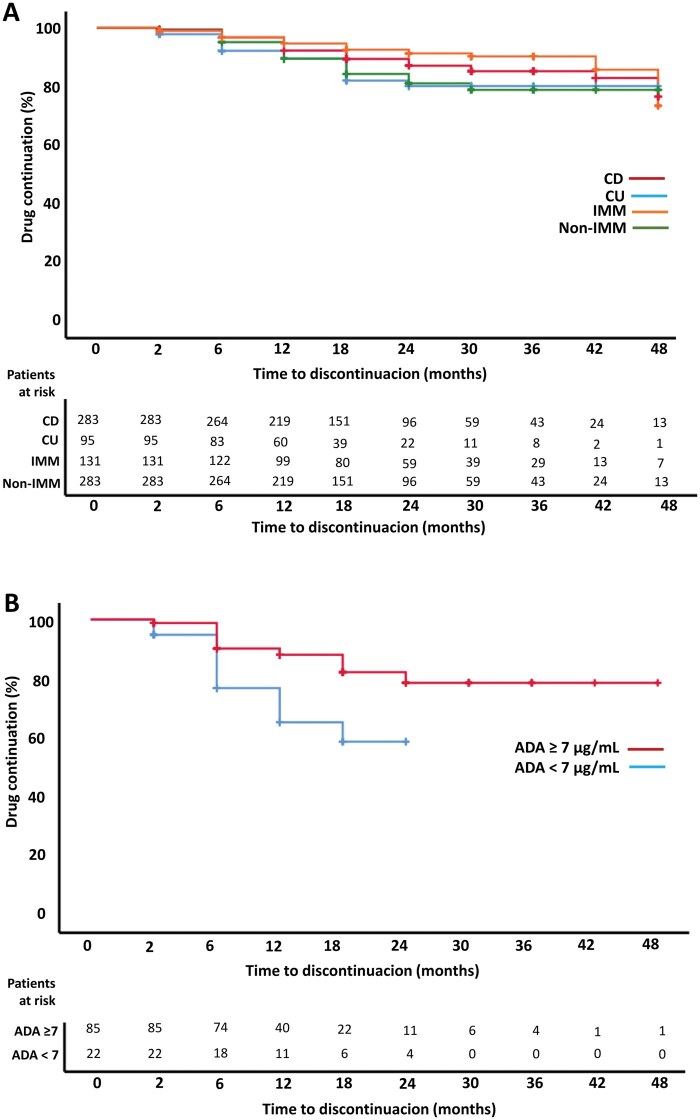
Kaplan-Meier curves of time to ADA biosimilar discontinuation in A) CD, UC, IMM, and non-IMM treated patients, and in B) patients with post-induction ADA levels ≥7 μg/mL or <7 μg/mL.

Drug persistence was significantly higher in CD patients than in UC patients (Figure 2A, *P* = .012). Although the proportion of patients without remission, or with SNR or PNR was numerically higher in UC patients, the differences were not significant (Table 2).

Drug persistence was also significantly higher in the group of patients treated with IMM (Figure 2A, *P* = .001). Similarly, patients treated with IMM showed a higher clinical remission rate at the end of follow-up (72.5% vs 65.4%, Table 2). The proportion of ­patients with SNR or PNR were numerically higher in non-IMM treated patients, although there was no significant difference (Table 2). Finally, dose intensification was performed in 35 (9.1%) patients during follow-up. Treatment intensification occurred more frequently in patients with UC (13.7% vs 7.4%, *P* = .065) and those who were not treated with IMM (12.3% vs 3.1%, *P* = .003).

### ADA trough levels and anti-drug antibodies

ADA levels were available in 107 and 225 patients, respectively. Median trough levels were 12.0 (8.4-17.5) µg/mL and 10.4 (6.7-13.0) µg/mL, respectively. Patients with post-induction ADA levels ≥ 7 μg/mL showed a higher drug persistence (Figure 2B, 76.5% vs 50.0%, *P* = .002). At week 8, ADA levels ≥ 7 μg/mL also showed a higher rate of clinical remission at the end of follow-up (63.5% vs 22.7%, *P* < .001). Additionally, the percentage of patients that maintained clinical remission at the end of follow-up was higher in this group of patients (78.4% vs 50.0%, *P* = .126). Anti-drug antibodies were evaluated in 101 patients after induction and in 220 patients during the follow-up period. The resulting positivity rates were 3.0% and 6.8%, respectively. Additionally, we found that the presence of anti-ADA antibodies was significantly associated with loss of response (*P* < .001), as 2.9% of patients who did not respond had detectable antibodies, compared to only 0.5% of responders. However, no association was found between anti-ADA antibodies and adverse events; in fact, no patient who experienced an adverse event tested positive for anti-ADA antibodies.

### Safety

In total, 30 (7.8%) patients experienced an AE (Table 4). The group treated with MSB11022 had the highest incidence of AEs, although this was not significantly different (Table 4). The percentage of AEs was quite similar in the other groups. Skin reactions were the most common AE (2.3%), with similar frequencies across all five biosimilars (Table 4). Opportunistic infections occurred in a total of 4 patients (1.4%): 2 in the GP2017 group, 1 in the MSB11022 group, and 1 in the ABP501 group. A total of 3 malignancies were reported in the GP2017 (1 cutaneous T-cell lymphoma, 0.4%) and ABP501 (1 bladder cancer and 1 supraglottic carcinoma, 2.5%) groups, with no deaths (Table 4). Finally, a total of 5 patients (1.3%) required hospitalization due to AEs. Furthermore, we did not find any significant association with IMM co-therapy (0.7).

**Table 4:** AEs categorized by the type of biosimilar used.

	Total	GP2017	ABP501	SB5	MSB11022	FKB327	*P* value^c^
	(*N* = 383)	(*N* = 230)	(*N* = 82)	(*N* = 19)	(*N* = 37)	(*N* = 15)	
**Total, *n* (%)**	30 (7.8)	16 (7.0)	7 (8.5)	1 (5.3)	5 (13.5)	1 (6.7)	.701
**Type, *n* (%)**							
** Skin reactions^a^**	9 (2.3)	6 (2.6)	1 (1.2)	1 (5.3)	1 (2.7)	-	-
** Infections^b^**	6 (1.6)	4 (1.7)	1 (1.2)	-	2 (5.4)	-	-
** Neoplasia**	3 (0.8)	1 (0.4)	2 (2.5)	-	-	-	-
** Cardio-respiratory events**	4 (1.0)	1 (0.4)	1 (1.2)	-	2 (5.4)	-	-
** Edema**	2 (0.5)	2 (0.9)	-	-	-	-	-
** Others**	6 (1.6)	2 (0.9)	2 (2.5)	-	-	1 (6.7)	-

a Skin reactions included 1 (3.4%) injection site reaction and psoriasiform lesions (26.7%).

b Infections included opportunistic infections (13.4%) and respiratory infections (6.7%).

c The *P*-value resulted from comparing the five biosimilars.

## Discussion

Our study shows that ADA biosimilars led to significant clinical remission in IBD patients who had not previously received biologics. Patients with CD, those treated with IMM, and those with post-induction ADA levels ≥ 7 μg/mL showed significantly higher drug persistence. The incidence of AEs was low, indicating the safety of ADA biosimilars. Furthermore, the comparison of the five available ADA biosimilars did not reveal any statistical differences in terms of effectiveness and safety.

This is the largest study to date evaluating the effectiveness of ADA biosimilars in bio-naive patients. To the best of our knowledge, there are two small studies that have assessed the clinical performance of ADA biosimilars in patients who have not previously received biologic treatment.^21^^,^^24^ These studies reported clinical remission rates of 79.6% and 58.6%,^21^^,^^24^ which are similar to the results found in this study (63.8% after induction and 67.9% at the end of the follow-up). Additionally, our findings align with those observed in patients previously exposed to other anti-TNF agents or biologics.^15^^,^^21^

The results of our study indicate that drug persistence was high (70.2%), exceeding the persistence rates reported for the original drug.^25^ This outcome is in line with those observed in previous studies involving different ADA biosimilars^15^^,^^16^ r7einforcing the similarity between biosimilars and the original drug. Furthermore, the cohort of patients included in this study has a relatively short disease progression time of 3.4 (0.8-10.0) years, which may explain the positive outcomes achieved. This result corroborates previous studies indicating that initiating biologic treatment at an early stage of IBD is associated with enhanced efficacy, particularly in CD.^26^

Another important finding is that combination therapy using IMM can increase the persistence of ADA biosimilar treatment. We found a higher persistence rate for IMM patients in comparison with patients not treated with these drugs (74.8% vs 67.9%). This finding is consistent with previous findings in IBD patients that combination use of IMM increased the persistence of biologic treatment.^25^^,^^27^ These findings suggest that continuing combination therapy with IMM may be beneficial for maintaining biosimilar persistence. On the other hand, a reason to explain this finding should be IMM could result in an anti-inflammatory effect and a decreased immunogenicity, which would lead to a reduction in loss of response and greater persistence.^25^ We found patients with post-induction ADA levels ≥ 7 μg/mL showed a higher drug persistence rate and higher clinical remission at the end of follow-up. Several studies have been published on the optimal ADA level to achieve clinical, endoscopic, and histological remission. These levels vary according to treatment goals.^28^ In this way, the optimal therapeutic cut-off point ranges from 4.5 to 12 μg/mL, where ADA levels are associated with adequate clinical follow-up of the disease during maintenance therapy.^29^ Our study confirms the association between serum ADA levels and clinical outcomes in the treatment of IBD. Therefore, we emphasize the importance of monitoring serum ADA levels to ensure the efficacy of biosimilar therapy and prevent loss of response in IBD patients.

A significant difference was found between patients with UC and CD. In our study, the persistence rate of UC patients was lower than those for the CD patients (71.4% vs 66.3%). This is consistent with the results reported by Chen et al.,^25^ which indicated that UC patients had a higher risk of stopping biologic treatment than patients with CD. This finding could be attributed to differences in disease pathophysiology and response to anti-TNF therapy, as well as potentially higher immunogenicity rates in UC, as ­previously reported by Chen et al.^25^ Additionally, previous studies have shown a poorer efficacy of both ADA biosimilars and originator in patients with UC.^24^^,^^30^ These insights highlight the importance of careful monitoring in UC patients and support considering combination therapy and personalized follow-up strategies to optimize treatment outcomes.

Only 10.6% of patients experienced an AE during follow-up, which is consistent with previously reported rates^19^^,^^21^ and with the rate of AEs commonly reported for the ADA originator.^31^^,^^32^ The incidence of AEs was low for all five ADA biosimilars in our population, with only 3 patients requiring hospitalization. We did not find any significant association between AE occurrence and IMM co-therapy (*P* = .7) or the presence of anti-drug antibodies. Furthermore, the number of AEs was too low to allow meaningful stratification analyses (eg, timing of onset or severity) or to determine whether malignancies were related to treatment. Overall, the results of the present study are consistent with those evaluating the safety of ADA biosimilars and confirm the safety of these drugs in real life.

The comparable effectiveness and safety of all five ADA biosimilars observed in our study has significant implications for both clinical practice and healthcare economics. Since no significant differences were found among the biosimilars, clinicians may prioritize factors such as cost, local availability, and tendering processes without compromising patient outcomes. This finding supports the rationale for choosing the most economically advantageous option, thereby maximizing cost savings for healthcare systems while maintaining high standards of care. Moreover, the comparable efficacy among biosimilars encourages their interchangeability, promoting broader access to biologic therapies and potentially reducing barriers to early treatment initiation in IBD. These results, therefore, reinforce the value of biosimilars as an effective and sustainable alternative to the originator product, aligning with the goals of both clinical effectiveness and financial stewardship in IBD management.

Our study has several strengths. First, it includes the largest sample size to evaluate the effectiveness of ADA biosimilars in biologic-naive IBD patients. Secondly, it has a long-term follow-up of over 12 months, providing more robust evidence compared to previous studies with shorter follow-up. Additionally, it is a real-life multicenter study that reflects clinical practice regarding the use of ADA biosimilars. Another strength of this study is that it compared all ADA biosimilars available for the first time.

However, it is important to acknowledge certain limitations. Due to the retrospective design of the study, there is a lack of follow-up data. Additionally, the data were not directly compared with that of the ADA originator. Instead, an indirect comparison of effectiveness was made with the originator studies. An additional limitation of our study is the predominance of GP2017 prescriptions (60.2%), which was largely due to local hospital pharmacy policies and supply logistics rather than clinical factors. While this imbalance could limit the generalizability of comparative findings, it also aligns with the biosimilar most frequently prescribed in our setting, thus reflecting real-world practice and providing relevant information on the most commonly used agent in our environment. Another limitation of our study is the absence of a multivariate analysis to identify independent predictors of treatment persistence. Given the retrospective design and real-world setting, we chose not to perform this analysis to avoid confounding, but future prospective studies should address this limitation. Finally, the retrospective nature and differences in local practices meant that endoscopic data were not systematically captured. Future research should prioritize incorporating objective measures like endoscopy data to provide a more complete assessment of treatment outcomes.

Further studies evaluating biosimilars in IBD patients naive to biologics are needed to provide a more comprehensive comparison with the data obtained in this study. We did not include data on endoscopic assessment, so mucosal healing rates could not be evaluated. Furthermore, treatment modifications may be made at the discretion of the responsible clinicians, which were not standardized. Although it is true that this reflects real-world practice, enabling direct translation of results into daily clinical practice.

## Conclusion

In conclusion, our findings demonstrate that ADA biosimilars are safe and effective in a real-life population of bio-naive patients with IBD. Drug persistence was significantly higher in patients with CD treated with IMM and with post-induction ADA levels ≥ 7 μg/mL. Furthermore, the five approved ADA biosimilars showed a similar level of effectiveness and safety. Overall, our study provides valuable insights that confirm the usefulness of ADA biosimilars in monitoring IBD patients.

## Data Availability

Data not publicly available.

## References

[1] Ng SC , Shi HY, Hamidi N, et al Worldwide incidence and prevalence of inflammatory bowel disease in the 21^st^ century: a systematic review of population-based studies. Lancet. 2017;390:2769-2778. 29050646 10.1016/S0140-6736(17)32448-0

[2] Graham DB , Xavier RJ. Pathway paradigms revealed from the genetics of inflammatory bowel disease. Nature. 2020;578:527-539. 32103191 10.1038/s41586-020-2025-2 PMC7871366

[3] Kumar A , Yassin N, Marley A, et al Crossing barriers: the burden of inflammatory bowel disease across Western Europe. Therap Adv Gastroenterol. 2023;16:17562848231218615. 10.1177/17562848231218615 PMC10748558 38144422

[4] Lamb CA , Kennedy NA, Raine T, et al; IBD Guidelines eDelphi Consensus Group. British society of gastroenterology ­consensus guidelines on the management of inflammatory bowel disease in adults. Gut. 2019;68:s1-106. 31562236 10.1136/gutjnl-2019-318484 PMC6872448

[5] Herrlinger KR , Stange EF. Twenty-five years of biologicals in IBD: what′s all the hype about? J Intern Med. 2021;290:806-825. 34128571 10.1111/joim.13345

[6] Van Der Valk ME , Mangen MJJ, Leenders M, et al; COIN Study Group and the Dutch Initiative on Crohn and Colitis. Healthcare costs of inflammatory bowel disease have shifted from hospitalisation and surgery towards anti-TNFα therapy: results from the COIN study. Gut. 2014;63:72-79. 23135759 10.1136/gutjnl-2012-303376

[7] Lawton J , Achit H, Pouillon L, et al Cost-of-illness of inflammatory bowel disease patients treated with anti-tumour necrosis factor: a French large single-centre experience. United European Gastroenterol J. 2019;7:908-913. 10.1177/2050640619853448 PMC6683635 31428415

[8] Alulis S , Vadstrup K, Olsen J, et al The cost burden of Crohn’s disease and ulcerative colitis depending on biologic treatment status—a Danish register-based study. BMC Health Serv Res. 2021;21:836. 34407821 10.1186/s12913-021-06816-3 PMC8371832

[9] Argollo M , Fiorino G, Gilardi D, et al Biosimilars of adalimumab in inflammatory bowel disease: are we ready for that? Curr Pharm Des. 2019;25:7-12. 30864505 10.2174/1381612825666190312113610

[10] Park W , Hrycaj P, Jeka S, et al A randomised, double-blind, multicentre, parallel-group, prospective study comparing the pharmacokinetics, safety, and efficacy of CT-P13 and innovator infliximab in patients with ankylosing spondylitis: the PLANETAS study. Ann Rheum Dis. 2013;72:1605-1612. 23687259 10.1136/annrheumdis-2012-203091 PMC3786643

[11] Yoo DH , Hrycaj P, Miranda P, et al A randomised, double-blind, parallel-group study to demonstrate equivalence in efficacy and safety of CT-P13 compared with innovator infliximab when coadministered with methotrexate in patients with active rheumatoid arthritis: the PLANETRA study. Ann Rheum Dis. 2013;72:1613-1620. 23687260 10.1136/annrheumdis-2012-203090 PMC3786641

[12] Buchner AM , Schneider Y, Lichtenstein GR. Biosimilars in inflammatory bowel disease. Am J Gastroenterol. 2021;116:45-56. 33110013 10.14309/ajg.0000000000000844

[13] Papp K , Bachelez H, Costanzo A, et al Clinical similarity of biosimilar ABP 501 to adalimumab in the treatment of patients with moderate to severe plaque psoriasis: a randomized, double-blind, multicenter, phase III study. J Am Acad Dermatol. 2017;76:1093-1102. 28291552 10.1016/j.jaad.2016.12.014

[14] Cohen S , Genovese MC, Choy E, et al Efficacy and safety of the biosimilar ABP 501 compared with adalimumab in patients with moderate to severe rheumatoid arthritis: a randomised, double-blind, phase III equivalence study. Ann Rheum Dis. 2017;76:1679-1687. 28584187 10.1136/annrheumdis-2016-210459 PMC5629940

[15] Tapete G , Bertani L, Pieraccini A, et al Effectiveness and safety of nonmedical switch from adalimumab originator to SB5 biosimilar in patients with inflammatory bowel diseases: twelve-month follow-up from the TABLET registry. Inflamm Bowel Dis. 2022;28:62-69. 33570142 10.1093/ibd/izab027

[16] Derikx LAAP , Dolby HW, Plevris N, et al Effectiveness and safety of adalimumab biosimilar SB5 in inflammatory bowel disease: outcomes in originator to SB5 switch, double biosimilar switch and Bio-Naïve SB5 observational cohorts. J Crohns Colitis. 2021;15:2011-2021. 34089587 10.1093/ecco-jcc/jjab100 PMC8684477

[17] Lukas M , Malickova K, Kolar M, et al Switching from originator adalimumab to the biosimilar sb5 in patients with inflammatory bowel disease: short-term experience from a single tertiary clinical Centre. J Crohns Colitis. 2020;14:915-919. 31905382 10.1093/ecco-jcc/jjaa001

[18] Casanova MJ , Nantes Ó, Varela P, et al Real-world outcomes of switching from adalimumab originator to adalimumab biosimilar in patients with inflammatory bowel disease: the ADA-SWITCH study. Aliment Pharmacol Ther. 2023;58:60-70. 37089065 10.1111/apt.17525

[19] Macaluso FS , Cappello M, Busacca A, et al; Sicilian Network for Inflammatory Bowel Disease (SN-IBD). SPOSAB ABP 501: a Sicilian prospective observational study of patients with inflammatory bowel disease treated with adalimumab biosimilar ABP 501. J Gastroenterol Hepatol. 2021;36:3041-3049. 34152636 10.1111/jgh.15590

[20] Wang F , Li X, Shi Y, et al Efficacy and safety of adalimumab biosimilar (HS016) in inflammatory bowel disease from the real-world study. Front Pharmacol. 2023;14:1259183. 37908975 10.3389/fphar.2023.1259183 PMC10613675

[21] Tursi A , Mocci G, Allegretta L, et al Comparison of performances of adalimumab biosimilars SB5, ABP501, GP2017, and MSB11022 in treating patients with inflammatory bowel diseases: a real-life, multicenter, observational study. Inflamm Bowel Dis. 2023;29:376-383. 35579320 10.1093/ibd/izac092

[22] Best WR. Predicting the Crohn’s disease activity index from the Harvey-Bradshaw index. Inflamm Bowel Dis. 2006;12:304-310. 16633052 10.1097/01.MIB.0000215091.77492.2a

[23] Lewis JD , Chuai S, Nessel L, et al Use of the noninvasive components of the Mayo score to assess clinical response in ulcerative colitis. Inflamm Bowel Dis. 2008;14:1660-1666. 18623174 10.1002/ibd.20520 PMC2597552

[24] Mocci G , Cingolani A, Orrù G, et al Adalimumab biosimilar ABP 501 is equally effective and safe in long-term management of inflammatory bowel diseases patients when used as first biologic treatment or as replace of the ADA originator for a non-medical reason. Front Gastroenterol. 2023;2:1218228. 10.3389/fgstr.2023.1218228 PMC12952366 41821801

[25] Chen C , Hartzema AG, Xiao H, et al Real-world pattern of biologic use in patients with inflammatory bowel disease: treatment persistence, switching, and importance of concurrent immunosuppressive therapy. Inflamm Bowel Dis. 2019;25:1417-1427. 30839057 10.1093/ibd/izz001

[26] Ben-Horin S , Novack L, Mao R, et al Efficacy of biologic drugs in short-duration versus long-duration inflammatory bowel disease: a systematic review and an individual-patient data meta-analysis of randomized controlled trials. Gastroenterology. 2022;162:482-494. 34757139 10.1053/j.gastro.2021.10.037

[27] Chanchlani N , Lin S, Bewshea C, et al; PANTS Consortium. Mechanisms and management of loss of response to anti-TNF therapy for patients with Crohn’s disease: 3-year data from the prospective, multicenter PANTS cohort study. Lancet Gastroenterol Hepatol. 2024;9:521-538. 38640937 10.1016/S2468-1253(24)00044-X

[28] Cheifetz AS , Abreu MT, Afif W, et al A comprehensive literature review and expert consensus statement on therapeutic drug monitoring of biologics in inflammatory bowel disease. Am J Gastroenterol. 2021;116:2014-2025. 34388143 10.14309/ajg.0000000000001396 PMC9674375

[29] Hinojosa J , Muñoz F, Martínez-Romero GJ. Relationship between serum adalimumab levels and clinical outcome in the treatment of inflammatory bowel disease. Dig Dis. 2019;37:444-450. 31039560 10.1159/000499870

[30] Wasserbauer M , Hlava S, Drabek J, et al Adalimumab biosimilars in the therapy of Crohn′s disease and ulcerative colitis: prospective multicentric clinical monitoring. PLoS One. 2022;17:e0271299. 35939424 10.1371/journal.pone.0271299 PMC9359532

[31] Tursi A , Elisei W, Faggiani R, et al Effectiveness and safety of adalimumab to treat outpatient ulcerative colitis: a real-life multicenter, observational study in primary inflammatory bowel disease centers. Medicine (Baltimore). 2018;97:e11897. 30142791 10.1097/MD.0000000000011897 PMC6112877

[32] Moćko P , Kawalec P, Pilc A. Safety profile of biologic drugs in the therapy of Crohn disease: a systematic review and network meta-analysis. Pharmacol Rep. 201;68:1237-1243. 27686963 10.1016/j.pharep.2016.07.013

